# Stepped care targeting psychological distress in head and neck cancer and lung cancer patients: which groups specifically benefit? Secondary analyses of a randomized controlled trial

**DOI:** 10.1007/s00520-019-04714-3

**Published:** 2019-03-27

**Authors:** Femke Jansen, Birgit I. Lissenberg-Witte, Anna M. H. Krebber, Pim Cuijpers, Remco de Bree, Annemarie Becker-Commissaris, Egbert F. Smit, Annemieke van Straten, Guus M. Eeckhout, Aartjan T. F. Beekman, C. René Leemans, Irma M. Verdonck-de Leeuw

**Affiliations:** 1 Department of Clinical, Neuro and Developmental Psychology, Amsterdam Public Health Research Institute, Vrije Universiteit Amsterdam, Amsterdam, The Netherlands; 2 Department of Otolaryngology-Head and Neck Surgery, Cancer Center Amsterdam (CCA), Amsterdam UMC, VU University Medical Center, Amsterdam, The Netherlands; 3 Department of Epidemiology and Biostatistics, Amsterdam UMC, VU University Medical Center, Amsterdam, The Netherlands; 4 UMC Utrecht Cancer Center, Utrecht, The Netherlands; 5 Department of Pulmonary Diseases, Amsterdam UMC, VU University Medical Center, Amsterdam, The Netherlands; 6 Department of Psychiatry, Amsterdam UMC, VU University Medical Center, Amsterdam, The Netherlands

**Keywords:** Moderators, Psychosocial intervention, Head and neck cancer, Anxiety, Depression, Distress

## Abstract

**Purpose:**

Stepped care (SC), consisting of watchful waiting, guided self-help, problem-solving therapy, and psychotherapy/medication is, compared to care-as-usual (CAU), effective in improving psychological distress. This study presents secondary analyses on subgroups of patients who might specifically benefit from watchful waiting, guided self-help, or the entire SC program.

**Methods:**

In this randomized controlled trial, head and neck and lung cancer patients with distress (*n* = 156) were randomized to SC or CAU. Univariate logistic regression analyses were performed to investigate baseline factors associated with recovery after watchful waiting and guided self-help. Potential moderators of the effectiveness of SC compared to CAU were investigated using linear mixed models.

**Results:**

Patients without a psychiatric disorder, with better psychological outcomes (HADS: all scales) and better health-related quality of life (HRQOL) (EORTC QLQ-C30/H&N35: global QOL, all functioning, and several symptom domains) were more likely to recover after watchful waiting. Patients with better scores on distress, emotional functioning, and dyspnea were more likely to recover after guided self-help. Sex, time since treatment, anxiety or depressive disorder diagnosis, symptoms of anxiety, symptoms of depression, speech problems, and feeling ill at baseline moderated the efficacy of SC compared to CAU.

**Conclusions:**

Patients with distress but who are relatively doing well otherwise, benefit most from watchful waiting and guided self-help. The entire SC program is more effective in women, patients in the first year after treatment, patients with a higher level of distress or anxiety or depressive disorder, patients who are feeling ill, and patients with less speech problems.

**Trial:**

NTR1868.

## Background

In the last decades, a wide range of psychosocial interventions has been developed targeting symptoms of psychological distress (i.e., anxiety and depression) in cancer patients [1–6]. These interventions differ in format (e.g., individualized or group intervention), type (e.g., self˗help or face-to-face), intensity, and duration. Stepped care (SC) models have been introduced as a method to organize psychosocial care. In SC models, patients are treated with low-intensive evidence-based interventions first, followed by more intensive interventions if symptoms do not resolve [2–5, 7]. It has been hypothesized that SC has the potential to improve the accessibility and efficacy of psychosocial care while limiting the burden on scarce healthcare resources [8].

So far, four studies have been performed on the efficacy of psychosocial SC interventions in cancer populations, including breast cancer [3], hematological cancer [5], head and neck cancer (HNC), lung cancer (LC) [4], and mixed cancer patient groups [2]. These interventions differed in care offered per step and study population targeted (i.e., all patients or patients with psychological distress only). These studies showed variable results [2–5]. The study targeting HNC and LC patients with psychological distress was found to be both effective and cost-effective [4, 9]. This study consisted of four steps, namely watchful waiting (step 1), guided self-help (step 2), face-to-face problem-solving therapy (step 3), and intensive psychological interventions and/or psychotropic medication (step 4) [10]. After step 1, 28% of all patients randomized to the SC group spontaneously recovered [4]. After step 2, approximately a third of all patients recovered [4], while after steps 3 and 4, although the sample size left was small, respectively 9 and 17% recovered. This resulted in an overall recovery rate of 55% in the SC group, while in the control group, in which care-as-usual (CAU) was provided, 29% recovered. In order to improve the efficacy of this SC program, more insight is needed into which patients specifically may benefit from steps 1 and 2 of the SC program, and which patients may not. This information is relevant to further tailor the SC program, for example by letting a patient skip a step in case this step is expected to be insufficiently effective.

Also, a detailed understanding is needed into (groups of) patients who specifically benefit from the SC program as a whole, compared to CAU. Previous studies on moderators of psychosocial care in cancer patients, in general, have consistently shown that patients with high levels of psychological distress specifically benefit from psychosocial care [6, 11, 12]. In addition, a previous individual patient data meta-analysis targeting cancer patients showed that psychosocial interventions were consistently more effective in younger patients and in those interventions in which psychotherapy was provided (compared to, e.g., psycho-education or coping skills training) [6]. For other potential moderators, so far, less consistent or only preliminary findings have been reported. A systematic review of 20 studies in cancer patients, in general, reported that patients with poorer quality of life, poorer interpersonal relationship, or lower self-efficacy specifically benefit from psychosocial interventions [13]. Also, patients with certain personality traits, such as low levels of optimism and lower levels of neuroticism, showed more beneficial results [13]. A study among HNC patients showed that a nurse-led psychosocial intervention was especially beneficial for patients who were married or living together, who had a poorer global quality of life, lower emotional functioning, or lower social functioning [14]. The SC program targeting HNC and LC patients was most beneficial for patients with a depressive or anxiety disorder, while for patients with symptoms of distress (but no depressive or anxiety disorder) the SC program was as effective as CAU [4]. More insight into other potential moderators, including sociodemographic, clinical, and quality of life factors, is warranted.

This study aimed to investigate for which (groups of) HNC and LC patients the SC program targeting psychological distress may be particularly effective. This insight was provided by (1) investigating baseline factors associated with recovery after step 1 (watchful waiting) and step 2 (guided self-help), and by (2) investigating potential moderators, including sociodemographic, clinical, and health-related quality of life (HRQOL) factors, of the efficacy of SC on psychological distress compared to CAU. The results of this study are relevant to further tailor care to the individual patient.

## Methods

### Study population

In this study, secondary analyses were performed using data of the randomized controlled trial on the efficacy of SC among HNC and LC patients [10]. Detailed information on the eligibility criteria, selection and randomization procedure, and sample size calculation is provided in the protocol and efficacy paper [4, 10]. In short, HNC and LC patients were asked to participate in the randomized controlled trial in case they were treated with curative intent at least 1 month earlier and had increased levels of symptoms of distress, anxiety, or depression, as defined by a Hospital Anxiety and Depression Scale (HADS) total score of > 14 or HADS-anxiety or HADS-depression subscale score of > 7. After providing informed consent and completing the first questionnaire, patients were randomized into either the SC or CAU group (1:1) by an independent person.

Medical ethical approval for this study was provided by the Medical Ethics Committee of VU University Medical Center. The study was registered in the Netherlands Trial Register (NTR1868).

### Stepped care and care-as-usual

The SC program consisted of four steps, namely watchful waiting (step 1), guided self-help via a book or the Internet (step 2), face-to-face problem solving therapy by a nurse (step 3), and intensive psychological interventions provided by a psychologist or psychiatrist and/or psychotropic medication (step 4) [10]. Patients stepped up to the next step if symptoms of psychological distress, anxiety, and/or depression did not resolve (i.e., HADS-total remained > 14 or HADS-depression or HADS-anxiety remained > 7). More information on the SC program is provided in the protocol paper [10]. The control group received CAU, which in most cases entailed no psychosocial care [4].

### Study measures

All patients were asked to complete a set of patient-reported outcome measures at six time points during the study period, namely at T0 baseline (before randomization), immediately after the intervention period or the control period of 4 months (T1), and 3, 6, 9, and 12 months after T1 (T2 to T5). The primary outcome of the study was the HADS [15, 16]. The HADS is a 14-item patient-reported outcome measure on symptoms of psychological distress, anxiety, and depression. The HADS total score ranges from 0 to 42, and the subscales from 0 to 21. A HADS total score > 14 or HADS subscale score > 7 was used as a cut-off for identifying persons with symptoms of psychological distress, anxiety or depression [17].

In conjunction with the HADS several other patient-reported outcome measures were collected, namely the European Organization for Research and Treatment of Cancer (EORTC) Quality of Life Questionnaire-Core 30-questions (QLQ-C30) [18, 19], the EORTC HNC-specific quality of life module (EORTC QLQ-H&N35) [20], the EORTC LC-specific quality of life module (QLQ-LC13) [21], and a patient-reported outcome measure for measuring patient satisfaction with care (EORTC IN-PATSAT32) [22]. For the analyses in the present study, only the EORTC QLQ-C30 and EORTC QLQ-H&N35 domains were used. All scores were linearly transformed to a 0 to 100 score. For functioning scales and the global quality of life scale, a higher score indicates better functioning or a better quality of life, while for all symptom scales, a higher score indicated worse symptoms.

Sociodemographic and clinical characteristics were also collected. Sociodemographic characteristics included age, sex, marital status, and work situation, which were collected via patient self-report. Clinical characteristics included tumor location, tumor stage, treatment, and time since treatment, which were collected from the hospital information system. Finally, a diagnostic telephone interview on psychiatric diagnoses, the Composite International Diagnostic Interview (CIDI), was assessed before randomization [23].

### Statistical analyses

All statistical analyses were performed using the Statistical Package for the Social Sciences version 22 (IBM Corp., Armonk, NY, USA). Descriptive statistics were generated for all sociodemographic and clinical characteristics.

To provide insight into factors associated with recovery from psychological distress after respectively step 1 (watchful waiting) and step 2 (guided self-help), exploratory univariate logistic regression analyses were performed. Only patients randomized to the SC program were included in these analyses. Factors which were investigated encompassed all sociodemographic and clinical characteristics and all baseline EORTC QLQ-C30, EORTC QLQ-H&N35 domains, and HADS outcomes. Factors associated with recovery after step 3 (problem-solving therapy) and step 4 (intensive psychological intervention or medication) were not performed due to the small sample size left in each of the groups. A *p* value < 0.05 was considered statistically significant.

To investigate potential moderators of the efficacy of SC compared to CAU on psychological distress (HADS-total) from baseline to 12 months follow-up, exploratory linear mixed model analyses were performed including fixed effects for time, group (SC or CAU), their two-way interaction, the potential moderator, and its two- and three-way interaction with group and time and a random intercept for subject. A significant (*p* < 0.05) three-way interaction was considered to indicate a difference of the efficacy of SC compared to CAU between groups with different scores on the investigated moderator. Post hoc linear mixed model analyses were performed to investigate the efficacy of SC on psychological distress compared to CAU, stratified for each subgroup of the found moderators separately. These stratified models included fixed effects for time, group and their two-way interaction, and a random intercept for subject. Potential moderators included all sociodemographic and clinical characteristics, having an anxiety or depressive disorder, and all baseline EORTC QLQ-C30, EORTC QLQ-H&N35 domains, and HADS outcomes. All EORTC QLQ-C30, EORTC QLQ-H&N35 domains, and HADS outcomes were dichotomized based on previously found cut-off scores [24, 25] or mean values found in the general population [26].

## Results

### Study population

**Fig. 1:**
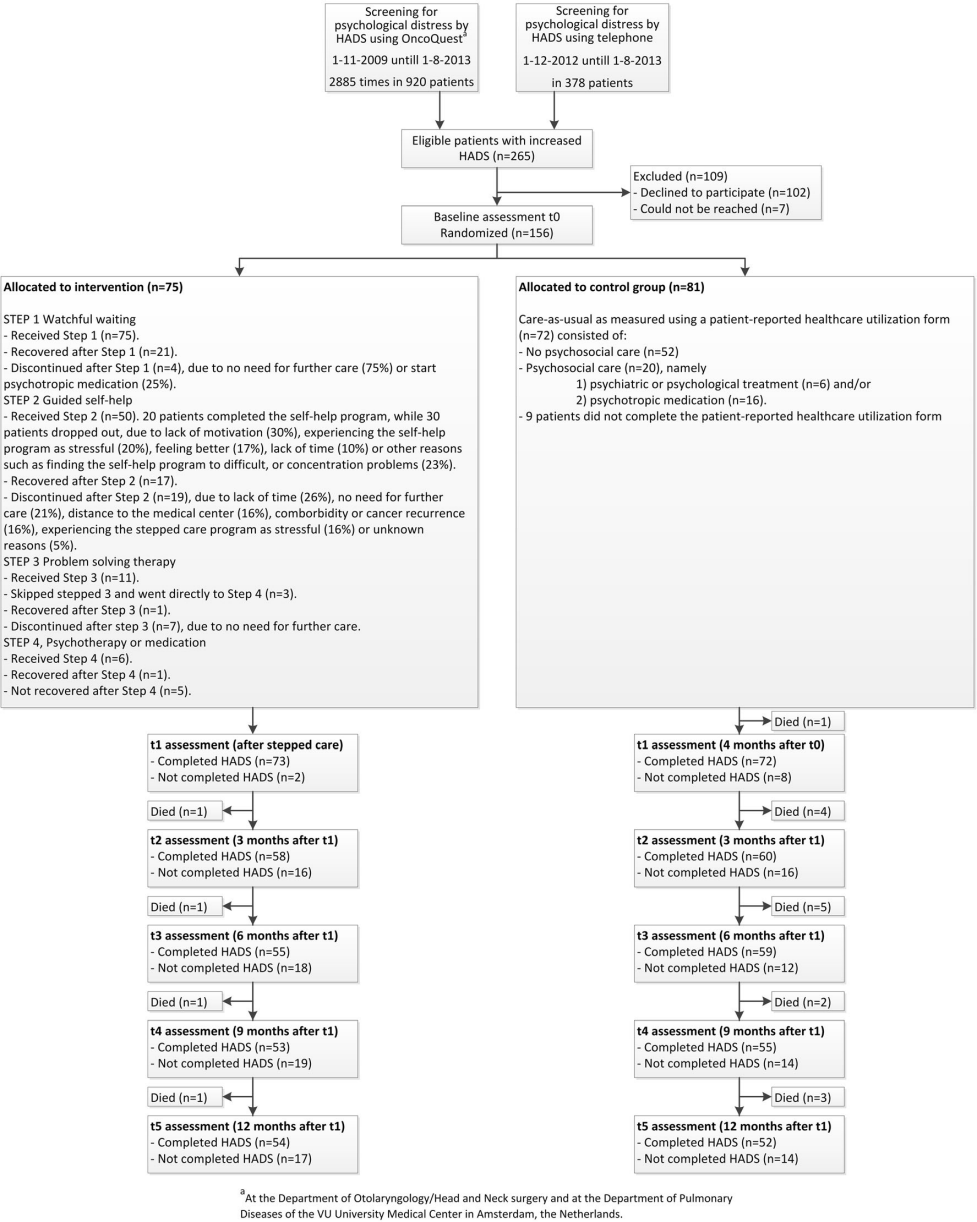
CONSORT flow diagram

In total, 81 patients were randomized to the CAU group and 75 patients to the SC group (Fig. 1). Patients randomized to SC were more likely to be alcohol dependent than patients in the CAU group (13 versus 4%, *p* = 0.030), as presented in Table 1. Also, patients in the intervention group scored better on depression (8.2 versus 9.5, *p* = 0.029), social functioning (70.3 versus 60.3, *p* = 0.026), and sexuality (39.6 versus 51.7, *p* = 0.040) at baseline.

**Table 1:** Baseline characteristics of patients in the care-as-usual and stepped care group

Characteristics	Care-as-usual	Stepped care
	*N* = 81	*N* = 75
Mean age, years (SD)	61.6 (10.0)	62.5 (8.7)
Sex		
Men	48 (59%)	47 (63%)
Women	33 (41%)	28 (37%)
Marital status		
Married/living with partner	52 (64%)	54 (72%)
Unmarried/divorced/widowed	29 (36%)	21 (28%)
Work situation		
Paid job	25 (31%)	23 (31%)
No paid job/retired	56 (69%)	52 (69%)
Tumor location		
Lip/oral cavity/oropharynx	46 (57%)	30 (40%)
Hypopharynx/larynx	19 (23%)	21 (28%)
Other head and neck cancers or lung	16 (20%)	24 (32%)
Tumor stage		
I–II	32 (41%)	32 (48%)
III–IV	47 (59%)	35 (52%)
Unknown	2	8
Treatment		
Single treatment	37 (46%)	39 (52%)
Combination treatment	44 (54%)	36 (48%)
Time since treatment		
<= 12 months	43 (53%)	39 (52%)
> 12 months	38 (47%)	36 (48%)
CIDI diagnosis (all)		
Yes	33 (41%)	27 (36%)
No	48 (59%)	48 (64%)
Anxiety or depressive disorder		
Yes	21 (26%)	14 (19%)
No	60 (74%)	61 (81%)
Nicotine dependence		
Yes	15 (19%)	12 (16%)
No	66 (81%)	63 (84%)
Alcohol dependence		
Yes	3 (4%)	10 (13%)*
No	78 (96%)	65 (87%)

*p < 0.05

### Watchful waiting (step 1) and guided self-help (step 2) of the stepped care program

Of all 75 patients randomized to SC, 21 patients (28%) recovered after the first step (watchful waiting). Of the remaining 54 patients, 50 patients continued with step 2 (guided self-help). Three patients discontinued after step 1 because they reported no need for further care and one patient discontinued because he/she started psychotropic medication. Of the 50 patients, 17 patients recovered (34%), and one patient was lost to follow-up.

Table 2 presents the results of factors associated with recovery after step 1 and step 2. Patients without a psychiatric diagnosis (all diagnoses) were more likely to recover after step 1 than patients with a psychiatric diagnosis (odds ratio (OR) = 4.80, 95% confidence interval (95%CI): 1.26–18.24). In addition, patients with higher (better) baseline scores on global quality of life and all functioning domains of the EORTC QLQ-C30 were more likely to recover after step 1, compared to patients with lower (worse) baseline scores (OR ranged from 1.02–1.05 per point increase). Patients with more problems regarding fatigue, pain, insomnia, swallowing, sticky saliva, and feeling ill (OR ranged from 0.96–0.98 per point increase), and worse scores on anxiety, depression, and distress (OR ranged 0.75–0.85 per point increase) were less likely to recover after step 1. Patients with better scores on emotional functioning (OR = 1.03 per point increase, 95%CI 1.002–1.06), dyspnea (OR = 0.97 per point increase, 95%CI 0.95–0.997), and distress (OR = 0.82 per point increase, 95%CI 0.69–0.98) were more likely to recover after step 2.

**Table 2:** Univariate logistic regression analyses of factors associated with recovery after watchful waiting (step 1) and guided self-help (step 2)

Characteristics	Treatment steps of the stepped care intervention^a^							
	Step 1 Watchful waiting				Step 2 Guided self-help			
	Recovered	Not recovered	OR	95%CI	Recovered^B^	Not recovered^B^	OR	95%CI
	*n* = 21	*n* = 54			*n* = 17	*n* = 32		
Mean age, years (SD)	61.6 (8.4)	62.9 (8.8)	0.98	0.93–1.04	61.8 (7.3)	62.8 (9.9)	0.99	0.92–1.06
Sex								
Men	13 (28%)	34 (72%)	1.00		11 (37%)	19 (63%)	1.00	
Women	8 (29%)	20 (71%)	1.05	0.37–2.96	6 (32%)	13 (68%)	0.80	0.24–2.70
Marital status								
Married/living with partner	15 (28%)	39 (72%)	1.00		12 (35%)	22 (65%)	1.00	
Unmarried/divorced/widowed	6 (29%)	15 (71%)	1.04	0.34–3.18	5 (33%)	10 (67%)	0.92	0.25–3.31
Work situation								
Paid job	7 (30%)	16 (70%)	1.00		5 (33%)	10 (67%)	1.00	
No paid job/retired	14 (27%)	38 (73%)	0.84	0.29–2.48	12 (35%)	22 (65%)	1.09	0.30–3.94
Tumor location								
Lip/oral cavity/oropharynx	8 (27%)	22 (73%)	1.00		6 (32%)	13 (68%)	1.00	
Hypopharynx/larynx	9 (43%)	12 (57%)	2.06	0.63–6.74	5 (45%)	6 (55%)	1.81	0.39–8.35
Other head and neck cancers or lung	4 (17%)	20 (83%)	0.55	0.14–2.11	6 (32%)	13 (68%)	1.00	0.26–3.93
Tumor stage								
I–II	8 (25%)	24 (75%)	1.00		7 (35%)	13 (65%)	1.00	
III–IV	12 (34%)	23 (66%)	1.57	0.54–4.53	9 (41%)	13 (59%)	1.29	0.37–4.50
Unknown	1	7			1	6		
Treatment								
Single treatment	8 (21%)	31 (79%)	1.00		9 (35%)	17 (65%)	1.00	
Combination treatment	13 (36%)	23 (64%)	2.19	0.78–6.15	8 (35%)	15 (65%)	1.01	0.31–3.27
Time since treatment								
<= 12 months	11 (28%)	28 (72%)	1.00		9 (35%)	17 (65%)	1.00	
> 12 months	10 (28%)	26 (72%)	0.98	0.36–2.69	8 (35%)	15 (65%)	1.01	0.31–3.27
CIDI diagnosis (all)								
Yes	3 (11%)	24 (89%)	1.00		7 (33%)	14 (67%)	1.00	
No	18 (38%)	30 (63%)	4.80	1.26–18.24*	10 (36%)	18 (64%)	1.11	0.34–3.66
Anxiety or depressive disorder								
Yes	1 (7%)	13 (93%)	1.00		5 (42%)	7 (58%)	1.00	
No	20 (33%)	41 (67%)	6.34	0.77–51.94	12 (32%)	25 (68%)	0.67	0.18–2.56
Nicotine dependence								
Yes	1 (8%)	11 (92%)	1.00		3 (30%)	7 (70%)	1.00	
No	20 (32%)	43 (68%)	5.12	0.62–42.40	14 (36%)	25 (64%)	1.31	0.29–5.87
Alcohol dependence								
Yes	1 (10%)	9 (90%)	1.00		2 (25%)	6 (75%)	1.00	
No	20 (31%)	45 (69%)	4.00	0.47–33.73	15 (37%)	26 (63%)	1.73	0.31–9.68
EORTC QLQ-C30	Mean (SD)	Mean (SD)	OR	95%CI	Mean (SD)	Mean (SD)	OR	95%CI
Global quality of life	71.0 (17.8)	54.6 (19.2)	1.05	1.02–1.08*	55.4 (16.1)	52.4 (20.7)	1.01	0.98–1.04
Physical functioning	81.0 (19.0)	68.4 (21.0)	1.03	1.004–1.07*	69.8 (20.7)	67.7 (20.2)	1.01	0.98–1.04
Role functioning	73.8 (26.1)	59.1 (27.3)	1.02	1.001–1.04*	62.7 (29.8)	55.9 (25.3)	1.01	0.99–1.03
Emotional functioning	70.2 (24.7)	52.6 (24.7)	1.03	1.01–1.06*	62.3 (23.6)	46.0 (23.9)	1.03	1.002–1.06*
Cognitive functioning	83.3 (21.7)	66.7 (27.3)	1.03	1.01–1.06*	73.5 (24.3)	60.8 (28.7)	1.02	0.99–1.04
Social functioning	81.0 (19.2)	66.0 (27.7)	1.03	1.002–1.05*	69.6 (25.2)	62.9 (29.1)	1.01	0.99–1.03
Fatigue	35.4 (25.2)	54.5 (24.9)	0.97	0.95–0.99*	51.0 (26.9)	58.8 (23.7)	0.99	0.96–1.01
Nausea Vomiting	4.8 (9.3)	12.3 (18.2)	0.96	0.91–1.01	6.9 (11.9)	15.1 (20.3)	0.96	0.92–1.01
Pain	20.6 (26.3)	38.4 (30.4)	0.98	0.96–0.997*	36.3 (25.8)	43.0 (33.5)	0.99	0.97–1.01
Dyspnea	25.4 (29.6)	32.7 (30.6)	0.99	0.97–1.01	19.6 (23.7)	41.1 (32.4)	0.97	0.95–0.997*
Insomnia	28.6 (30.3)	49.1 (37.9)	0.98	0.97–0.999*	35.3 (38.1)	58.1 (37.5)	0.98	0.97–1.000
Loss of appetite	17.5 (29.1)	33.3 (35.8)	0.98	0.97–1.002	21.6 (31.0)	42.0 (38.5)	0.98	0.97–1.002
Constipation	12.7 (22.3)	17.9 (26.0)	0.99	0.97–1.01	19.6 (29.0)	18.9 (25.8)	1.00	0.98–1.02
Diarrhea	4.8 (12.0)	18.2 (30.4)	0.97	0.94–1.003	23.5 (36.8)	18.3 (28.3)	1.01	0.99–1.02
Financial problems	20.1 (28.7)	15.9 (25.0)	0.99	0.98–1.01	19.6 (26.5)	22.6 (31.5)	1.00	0.98–1.02
EORTC QLQ-H&N35^c^								
Oral pain	20.0 (18.6)	33.0 (29.7)	0.98	0.96–1.003	25.5 (28.2)	39.2 (31.8)	0.98	0.96–1.01
Swallowing	11.7 (13.1)	34.4 (29.2)	0.96	0.93–0.99*	29.4 (28.0)	36.7 (29.9)	0.99	0.97–1.01
Senses problems	27.5 (31.7)	26.2 (25.2)	1.00	0.98–1.02	25.5 (25.8)	29.6 (25.9)	0.99	0.97–1.02
Speech problems	17.8 (18.5)	28.8 (26.2)	0.98	0.96–1.004	23.5 (22.9)	30.9 (27.8)	0.99	0.96–1.01
Trouble with social eating	20.0 (26.8)	32.7 (28.0)	0.98	0.96–1.003	25.5 (25.1)	37.0 (28.7)	0.98	0.96–1.01
Trouble with social contact	11.3 (15.3)	19.6 (19.2)	0.97	0.94–1.01	14.5 (17.7)	21.5 (18.3)	0.98	0.94–1.01
Sexuality	34.2 (39.9)	41.8 (32.2)	0.99	0.98–1.01	47.1 (32.9)	36.7 (30.4)	1.01	0.99–1.03
Teeth	21.7 (29.2)	22.2 (31.8)	1.00	0.98–1.02	7.8 (18.7)	25.6 (35.7)	0.98	0.95–1.003
Opening mouth	18.3 (27.5)	36.8 (37.2)	0.98	0.97–1.000	31.4 (41.6)	40.7 (36.2)	0.99	0.98–1.01
Dry mouth	43.3 (37.6)	55.6 (34.6)	0.99	0.98–1.01	47.1 (33.5)	64.1 (35.2)	0.99	0.97–1.004
Sticky saliva	25.0 (26.2)	46.3 (33.9)	0.98	0.96–0.996*	33.3 (26.4)	51.9 (35.0)	0.98	0.96–1.002
Coughing	30.0 (28.4)	36.7 (29.8)	0.99	0.97–1.01	29.4 (28.6)	39.5 (30.7)	0.99	0.97–1.01
Felt ill	13.3 (19.9)	35.4 (28.4)	0.97	0.94–0.99*	29.4 (26.0)	40.7 (29.7)	0.99	0.96–1.01
HADS								
Anxiety	7.3 (4.1)	10.1 (3.0)	0.76	0.64–0.92*	9.1 (2.7)	10.7 (3.1)	0.82	0.65–1.02
Depression	6.8 (3.9)	8.7 (3.5)	0.85	0.73–0.99*	7.7 (3.0)	9.3 (3.4)	0.85	0.70–1.04
Total	14.0 (4.1)	18.9 (4.9)	0.75	0.63–0.89*	16.8 (3.6)	20.0 (4.7)	0.82	0.69–0.98*

^a^Only factors associated with recovery after steps 1 and 2 are presented, as the total sample size in steps 3 and 4 was too small

^B^This analysis includes patients who did not recover after step 1 and who continued with step 2. Of the 54 patient who did not recover after step 1, 50 patients continued with step 2. One of these patients was lost to follow-up

^c^HNC patients only

*p < 0.05

### Moderators of the efficacy of stepped care on psychological distress compared to care-as-usual

**Fig. 2:**
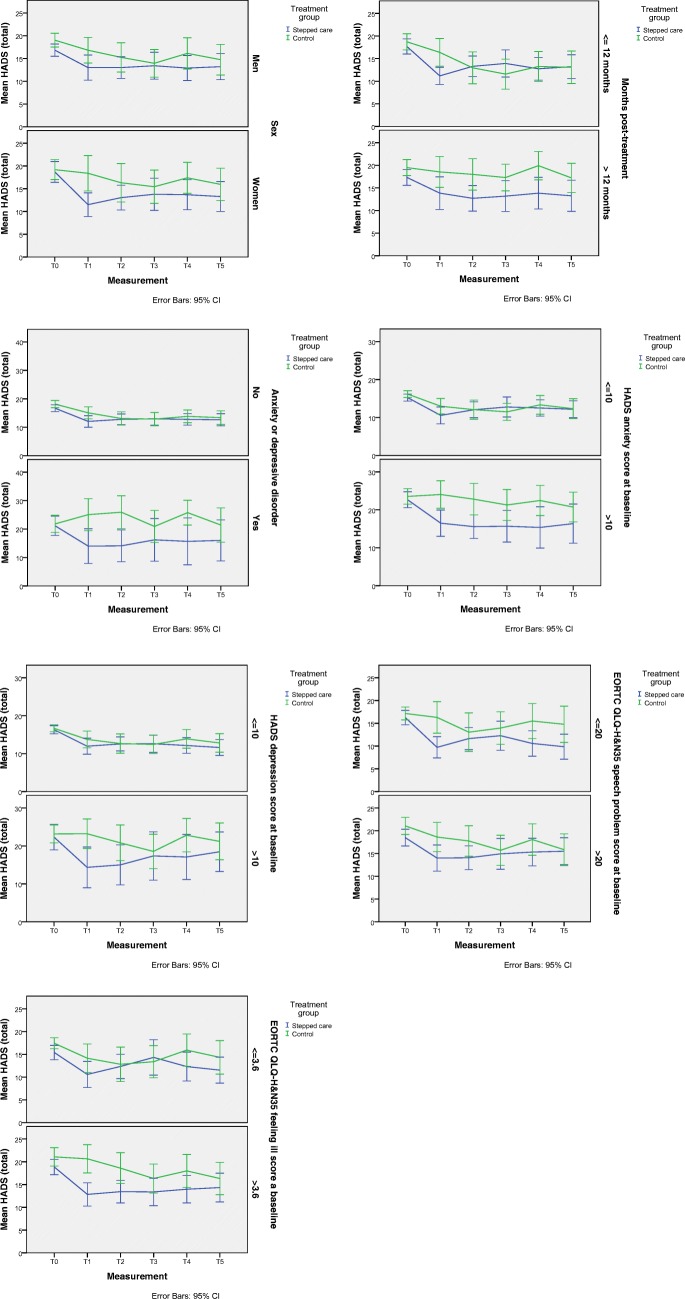
Moderators (HADS total)

Seven factors were found to significantly moderate the effect of SC on psychological distress compared to CAU (all showed a three-way interaction of *p* < 0.05), namely sex, time since oncological treatment, having an anxiety or depressive disorder (as also reported in our previous study [4]), symptoms of anxiety, symptoms of depression, speech problems, and feeling ill at baseline (Table 3 and Fig. 2). Post hoc analyses showed that SC was more effective in women (*p* = 0.002), patients in the first year after treatment (*p* < 0.001), patients with an anxiety or depressive disorder (*p* = 0.002), patients with worse score on anxiety (*p* = 0.002), depression (*p* = 0.002) and feeling ill (*p* < 0.001), and patients with better scores on speech problems (*p* < 0.001) at baseline, compared to CAU. In men (*p* = 0.14), patients longer than 1 year after treatment (*p* = 0.40), patients without an anxiety disorder (*p* = 0.18), patients with better scores on anxiety (*p* = 0.052), depression (p = 0.40) and feeling ill (*p* = 0.056), and worse scores on speech problems (*p* = 0.11), SC was as effective as CAU.

**Table 3:** Significant moderators of the effect of stepped care on psychological distress compared to care-as-usual

Moderator		Condition	N	Moderation analyses		Post hoc analyses	
				F (df)^1^ three-way interaction	*P* value three-way interaction	F (df)^1^ two-way interaction	*P* value two-way interaction
Sex	Men	Stepped care	47	2.328 (578.99)	0.041	1.684 (342.75)	0.14
		Care-as-usual	48				
	Women	Stepped care	28			3.985 (236.51)	0.002
		Care-as-usual	33				
Months since treatment	<= 12 months	Stepped care	39	2.869 (578.93)	0.014	5.288 (293.17)	< 0.001
		Care-as-usual	43				
	> 12 months	Stepped care	36			1.036 (285.54)	0.40
		Care-as-usual	38				
Anxiety or depression disorder	No	Stepped care	61	4.018 (580.63)	0.001	1.517 (467.32)	0.18
		Care-as-usual	60				
	Yes	Stepped care	14			4.058 (111.96)	0.002
		Care-as-usual	21				
HADS-anxiety	<= 10	Stepped care	52	3.093 (584.39)	0.009	2.219 (401.71)	0.052
		Care-as-usual	49				
	> 10	Stepped care	23			4.014 (181.44)	0.002
		Care-as-usual	32				
HADS-depression	<= 10	Stepped care	60	2.397 (580.67)	0.036	1.035 (426.21)	0.40
		Care-as-usual	51				
	> 10	Stepped care	15			3.880 (155.23)	0.002
		Care-as-usual	30				
EORTC QLQ-H&N35 speech problems	<= 20	Stepped care	32	2.619 (536.07)	0.024	4.966 (232.99)	< 0.001
		Care-as-usual	32				
	> 20	Stepped care	37			1.805 (303.33)	0.11
		Care-as-usual	43				
EORTC QLQ-H&N35 feeling ill	<= 3.6	Stepped care	28	2.392 (540.92)	0.037	2.186 (241.42)	0.056
		Care-as-usual	35				
	> 3.6	Stepped care	41			4.590 (299.78)	< 0.001
		Care-as-usual	41				

*N*, number of patients; *df*, degrees of freedom; *HADS*, Hospital Anxiety and Depression Scale; *EORTC QLQ-H&N35*, European Organization for Research and Treatment of Cancer head and neck cancer-specific quality of life module

^1^Numerator df = 5

## Discussion

This study aimed to provide insight into groups of HNC and LC patients for which the SC program, consisting of watchful waiting, guided self-help, face-to-face problem-solving therapy, and intensive psychological interventions and/or psychotropic medication, as steps with increasing intensity of treatment, may be particularly effective. It was found that patients with less impairments in functioning and symptoms at baseline benefitted from watchful waiting and guided self-help. Also, patients without a psychiatric diagnosis were more likely to recover after 2 weeks of watchful waiting, compared to patients with a psychiatric diagnosis. In addition, it was found that the SC program as a whole, compared to CAU, was especially effective in women, patients in the first year after treatment, patients with an anxiety or depressive disorder, patients with a worse baseline score on anxiety, depression and feeling ill, and patients with a better score on speech problems.

Especially patients with psychological distress but who are doing relatively well otherwise (without a psychiatric diagnosis and with less impairment in functioning and less symptoms) seem to benefit from watchful waiting as such. This may be explained by the fact that patients are screened on psychological distress at their follow-up visit. Reassurance after this visit that the cancer is in remission may have resulted in the diminishment of psychological distress [4]. It makes sense that patients who spontaneously recover are the patients with less impairments in functioning or symptoms. Of those patients who did not spontaneously recover, 34% recovered after guided self-help. Also, patients who benefitted from this step were doing relatively well (better scores on emotional functioning, dyspnea, and psychological distress). The findings on emotional functioning and psychological distress are consistent with the tenets of SC in which low-intensive treatment is expected to be beneficial in patients with lower level of symptoms, while more intensive treatments are saved for those patients with more serious symptoms.

When focusing on the SC program as a whole, we found that SC was, compared to CAU, especially beneficial in women, patients in the first year after treatment, patients who were doing relatively not so well, and patients with better scores on speech problems. The finding that the SC program was particularly effective in patients with an anxiety or depressive disorder or patients with worse anxiety and depression symptoms is in line with previous studies [6, 11, 12]. Our result that SC is especially effective in women is, however, in contrast to the results of a meta-analysis using individual patient data of mixed groups of cancer patients [6] and a nurse-led psychosocial intervention in patients with HNC, which found no moderating effect of sex [14]. Another meta-analysis among mixed groups of cancer patients, on the other hand, showed higher effects in men [27], but after excluding sex-specific cancers (e.g., breast and prostate) no statistically significant difference in effect was evident [27]. Further research is needed to investigate whether our finding that SC is especially beneficial in women can be replicated.

HRQOL outcomes, including global quality of life, emotional functioning, and social functioning, were not found to moderate the efficacy of SC in the present study, while these factors have been found to moderate the efficacy of a nurse-led psychosocial intervention among HNC patients [14]. This warrants further research on the moderating role of HRQOL on the efficacy of psychosocial interventions. The reason why patients with a better HRQOL score on speech problems may benefit more from SC may be that, especially for problem-solving therapy and intensive psychological interventions (steps 3 and 4), oral conversations are part of the psychosocial treatment. In case of speech problems, it may be difficult to express oneself, which might potentially impair the effect of such psychosocial interventions.

### Study limitations

A major limitation is that this study was not powered to perform these secondary exploratory analyses, therefore, the results should be interpreted with caution. Because of the low sample size, we also only performed univariate logistic regression analyses and did not investigate factors associated with recovery after steps 3 and 4. Moreover, only a few patients with LC participated in this study impairing the generalizability of the findings to this group of cancer patients.

### Clinical implications

This study provided important information to further tailor SC to the individual patient. Results showed that patients who benefitted from watchful waiting and guided self-help were those with less impairments in functioning and symptoms. Also, patients who recovered after 2 weeks of watchful waiting less often had a psychiatric diagnosis, compared to patients who did not recover. The SC program as a whole was especially effective in women, patients in the first year after treatment, patients with an anxiety or depressive disorder, patients with worse scores on anxiety, depression, and speech problems, and patients with better scores on feeling ill, as compared to CAU. This information can be used to further tailor SC, e.g., by skipping steps which are expected to be insufficiently effective to the individual patient in clinical practice.
